# Clinical Outcomes Among Working Adults Using the Health Integrator Smartphone App: Analyses of Prespecified Secondary Outcomes in a Randomized Controlled Trial

**DOI:** 10.2196/24725

**Published:** 2022-03-21

**Authors:** Stephanie Bonn, Gabriella Licitra, Rino Bellocco, Ylva Trolle Lagerros

**Affiliations:** 1 Clinical Epidemiology Division Department of Medicine Solna Karolinska Institutet Stockholm Sweden; 2 Department of Medical Epidemiology and Biostatistics Karolinska Institutet Stockholm Sweden; 3 Department of Statistics and Quantitative Methods University of Milano-Bicocca Milan Italy; 4 Center for Obesity, Academic Specialist Center Stockholm Health Services Stockholm Sweden

**Keywords:** adults, body composition, exercise, HbA1c, healthy lifestyle, metabolic health, mobile app, randomized controlled trial, smartphone

## Abstract

**Background:**

There is a need to find new methods that can enhance the individuals’ engagement in self-care and increase compliance to a healthy lifestyle for the prevention of noncommunicable diseases and improved quality of life. Mobile health (mHealth) apps could provide large-scale, cost-efficient digital solutions to implement lifestyle change, which as a corollary may enhance quality of life.

**Objective:**

Here we evaluate if the use of a smartphone-based self-management system, the Health Integrator app, with or without telephone counseling by a health coach, had an effect on clinical variables (secondary outcomes) of importance for noncommunicable diseases.

**Methods:**

The study was a 3-armed parallel randomized controlled trial. Participants were randomized to a control group or to 1 of 2 intervention groups using the Health Integrator app with or without additional telephone counseling for 3 months. Clinical variables were assessed before the start of the intervention (baseline) and after 3 months. Due to the nature of the intervention, targeting lifestyle changes, participants were not blinded to their allocation. Robust linear regression with complete case analysis was performed to study the intervention effect among the intervention groups, both in the entire sample and stratifying by type of work (office worker vs bus driver) and sex.

**Results:**

Complete data at baseline and follow-up were obtained from 205 and 191 participants, respectively. The mean age of participants was 48.3 (SD 10) years; 61.5% (126/205) were men and 52.2% (107/205) were bus drivers. Improvements were observed at follow-up among participants in the intervention arms. There was a small statistically significant effect on waist circumference (β=–0.97, 95% CI –1.84 to –0.10) in the group receiving the app and additional coach support compared to the control group, but no other statistically significant differences were seen. However, participants receiving only the app had statistically significantly lower BMI (β=–0.35, 95% CI –0.61 to –0.09), body weight (β=–1.08, 95% CI –1.92 to –0.26), waist circumference (β=–1.35, 95% CI –2.24 to –0.45), and body fat percentage (β=–0.83, 95% CI –1.65 to –0.02) at follow-up compared to the controls. There was a statistically significant difference in systolic blood pressure between the two intervention groups at follow-up (β=–3.74, 95% CI –7.32 to –0.16); no other statistically significant differences in outcome variables were seen.

**Conclusions:**

Participants randomized to use the Health Integrator smartphone app showed small but statistically significant differences in body weight, BMI, waist circumference, and body fat percentage compared to controls after a 3-month intervention. The effect of additional coaching together with use of the app is unclear.

**Trial Registration:**

ClinicalTrials.gov NCT03579342; https://clinicaltrials.gov/ct2/show/NCT03579342

**International Registered Report Identifier (IRRID):**

RR2-10.1186/s12889-019-6595-6

## Introduction

On the World Health Organization (WHO) list of the top 10 causes of death for 2020, three of the four top causes, ischemic heart disease, stroke, and chronic obstructive pulmonary disease, are all noncommunicable diseases strongly linked to lifestyle [1]. The etiology of such diseases includes lifestyle-related risk factors such as physical inactivity, unhealthy diet, and tobacco use. WHO estimates that almost 90% of premature deaths due to noncommunicable diseases occur in low- and middle-income countries.

Mobile health (mHealth), defined as “medical and public health practice supported by mobile devices, such as mobile phones, patient monitoring devices, personal digital assistants, and other wireless devices” is one approach to take global action for the prevention and control of noncommunicable diseases [2]. WHO has acknowledged the potential benefit of mHealth for public health and called for evidence of effectiveness [3].

The rapid evolution of information technology has made smartphones an integral part of daily life. Worldwide, 48% of people own a smartphone [4]; in Sweden, this number is 92% [5]. Furthermore, ownership and use are independent of socioeconomic status. This makes Sweden a suitable country to evaluate intervention research using smartphone technology, which later can be disseminated to other populations.

A number of studies have been conducted on the use of smartphone technology to implement and support lifestyle changes among persons with noncommunicable diseases such as diabetes [6,7]. A systematic review of the literature found that the most common topics for health promotion programs using mobile apps were diet and physical activity, and across the studies included, app users were more successful in terms of health outcomes [8]. A meta-analysis including more than 6000 participants in app-based mobile interventions for improving nutrition behaviors and nutrition-related health outcomes did not find any evidence that additional intervention components or number of behaviour change techniques included in the intervention mattered [9]. Contrasting these results, authors of another systematic review concluded that multicomponent interventions that combined the smartphone app with some other kind of intervention appeared more effective than stand-alone app interventions where the app was the only intervention component [10]. The use of additional health coaches complementing an app has been evaluated in lifestyle interventions targeting for example persons with diabetes [11-13]. While health coaches can help to set realistic goals and encourage when motivation fails, they are also less scalable. Whereas some studies have shown favorable results in the smartphone and coach-assisted groups compared to control groups [11,13], others could not find a significant difference between groups [12]. Clearly, a smartphone app available at any time is more convenient compared to scheduled face-to-face or group interventions, but it has not been convincingly documented if multicomponent smartphone-assisted lifestyle interventions, with or without health coaches, are effective in changing lifestyle behaviors.

Based on evidence from previous research on smartphone-assisted lifestyle interventions, we developed and built a new digital platform and an adjacent smartphone app for lifestyle change called the Health Integrator as part of a European collaboration granted by the European Institute of Innovation and Technology (EIT).

The Health Integrator core system consists of a home page, a smartphone app, and underlying supporting technical services and infrastructure. The platform was developed to be a collaboration tool for individuals in need of lifestyle change and their health coaches as a means to provide a personalized health improvement program. The program offers a variety of public, private, and community services for behavior change in different domains such as smoking, alcohol, physical activity, diet, stress, and sleep. These offers can be accessed through the Health Integrator smartphone app for those in need of lifestyle change who have a personal user account.

The aim of this study was to evaluate if use of the Health Integrator, offering a multicomponent lifestyle intervention for 3 months, with or without additional counseling by a health coach, had an effect on clinical variables (secondary outcomes) of importance for noncommunicable diseases such as BMI, waist circumference, and blood pressure in working adults and, more specifically, in office workers and bus drivers.

## Methods

### Study Design

The design of the Health Integrator study has been described in detail elsewhere [14]. In brief, the study was a 3-armed parallel randomized controlled trial with allocation 1:1:1, to one of two intervention arms or a control group. The active intervention was 3 months. Assessment of lifestyle behavioral factors and clinical variables was done before the start of the intervention (baseline) and after completed intervention at 3-month follow-up. Power was calculated based on the main outcome, change in general health perception [14], and the intervention was a priori planned to include at least 63 participants in each study arm. The intervention was described according to the CONSORT-EHEALTH checklist [15].

### Participants

Both men and women were eligible for participation and inclusion criteria were being aged 18 years or older, understanding Swedish well enough to understand the study aims, providing informed consent for participation, and having access and ability to use a smartphone.

Study participants were recruited from 4 companies: 2 companies with white-collar employees (ie, office workers) and 2 companies with blue-collar employees, (ie, bus drivers). After discussions with the offices of the human relations at the different companies, recruitment to the study was conducted in 2 different ways according to the wishes of the companies. White-collar employees got an email from the office of human relations with information about the study. Those who were interested in participating responded by email, and research personnel then received their email addresses. Bus drivers, on the other hand, whose email addresses were unknown to the employer, were informed by study personnel at the bus garages and asked in person to provide their private email. Thereafter, study personnel emailed detailed information about the study and a link to access the web-based baseline questionnaire to all employees interested in the study.

### Data Collection

After having completed the baseline questionnaire, all respondents were provided with a link to the Health Integrator system. They were asked to answer additional questions creating a health profile in the system and could thereafter schedule a time for the baseline meeting with the health coach. At this point, participants had not been randomized and the results from the health profile questions were not yet visible to the participant in the Health Integrator system.

All study participants meet with study personnel at baseline and again after 3-month follow-up. All participants received an accelerometer to assess physical activity during 7 consecutive days during the baseline visit, and body composition and blood pressure were measured on both visits. At baseline, results from the health profile were also unlocked for participants in the 2 intervention groups but not for participants in the control group. All participants also received a referral for analysis of glycated hemoglobin A_1c_ (HbA_1c_) and serum lipids. At the 3-month follow-up, only participants with an HbA_1c_ or serum lipids outside clinical reference values were given a second referral. Before the 3-month follow-up meeting, participants responded to the web-based questionnaire and the additional health profile questions again. Participants also responded to a final web-based questionnaire after 6 months.

### Randomization

Participants were randomized to 1 of 3 groups: intervention group A receiving the Health Integrator smartphone app and additional coach support, intervention group B receiving the Health Integrator smartphone app without additional coach support, and control group C did not receive the Health Integrator smartphone app or any coach support. Participants in the control group received access to the app after the active intervention had been completed at 3-month follow-up. Randomization was done in blocks of 6 by company and sex using a random allocation list generated by the first author using Stata (version 15.1, StataCorp LLC). Each new participant included in the study was continuously assigned to the next available randomly allocated group on the computer-generated list by the first author. Participants were randomized before the baseline meeting and informed about their allocation when meeting with study personnel at baseline measurements. Study personnel were not blinded to the allocation during the meeting but did not reveal the allocation to the participant until after performing baseline assessments. Due to the nature of the intervention, participants were not blinded to their allocation.

### Intervention

During the baseline meeting, participants that had been randomized to any of the 2 intervention groups (with and without additional coach support) downloaded the Health Integrator smartphone app. The app was compatible with both Android (version 4.1 and higher) and iOS (version 8 and higher). User satisfaction with the app was assessed at the end of the trial [16].

Results from the health profile were discussed with the health coach at the baseline meeting. The participant and the health coach decided on which lifestyle behavior to target based on the health profile. Thereby, each participant received a personalized intervention, customized based on their needs and goals. The intervention could target any of the following 6 areas: diet, physical activity, sleeping habits, stress, alcohol, or tobacco use.

Within the Health Integrator system, a number of different offers related to the different intervention areas were available. For example, a participant aiming to increase physical activity levels could choose between offers including, for example, other smartphone apps developed to promote physical activity specifically (eg, Runkeeper), to get a wrist support band to facilitate rehabilitation, receive a training pass at a local gym, or set the goal to take part in a race and have the fee paid for. There were in total 37 offers. The offers were free of charge for the participant.

Related to the target area, the health coach together with the participant identified and agreed on a suitable weekly goal, which was entered into the Health Integrator. This could, for example, be number of sessions of physical activity per week. To keep on track during the 3 months of active intervention, participants recorded if the weekly goal was met using an emoticon scale (ie, smiley faces) or by marking the number of days that the goal was met during the week. A reminder to record progression was sent out every Sunday at 9:20 PM. Those participants randomized to receive the Health Integrator smartphone app and additional health coach support had a scheduled telephone appointment every 4 weeks with the health coach. During these 30-minute sessions, participants were given personalized guidance and encouragement to support the lifestyle change in question. During these sessions, it was also possible to update or change the weekly goal.

### Control Group

During the active intervention for the intervention groups, the control group participants were not given any lifestyle recommendations nor were they aware of which offers were included in the app. Results from the baseline health profile were shown and discussed with the health coach at the 3-month follow-up (ie, after the active intervention). At that time, participants in the control group were offered to download the Health Integrator app and were given access to the same offers as the intervention groups.

### Outcome Measures

Self-reported data on civil status (having a partner vs not having a partner), smoking (yes/no), snuff use (yes/no), physical activity (<150 min/wk vs 150-300 min/wk vs ≥300 min/wk), and treatment for diabetes (yes/no), hypertension (yes/no), high serum lipids (yes/no), and diabetes risk using the Finnish Diabetes Risk Score (FINDRISC) were retrieved from the extensive web-based baseline questionnaire [17]. See Multimedia Appendix 1 for a complete list of study assessments in the trial.

Weight (kg), waist circumference (cm), body fat (percentage), and blood pressure (mm Hg) were measured by study personnel at baseline and follow-up after 3 months. Height (cm) was self-reported at baseline. BMI (kg/m^2^) was calculated based on measured weight and reported height. Body weight, waist circumference, and body fat percentage were analyzed separately for women and men.

At baseline, all participants received a referral for analysis of HbA_1c_ (mmol/mol), total cholesterol (mmol/L), apolipoprotein A1 (g/L), and apolipoprotein B (g/L).

### Ethical Considerations

The study was approved by the regional ethical review board in Stockholm, Sweden (2018/411-31 and 2018/1038-32). The trial was registered at ClinicalTrials.gov [NCT03579342]. Eligible participants were required to give their informed consent prior to responding to the baseline questionnaire. After reading an introductory screen displaying information about the study, participants were required to consent to participate in order to continue to the questionnaire. At the baseline meeting, participants also gave their written informed consent.

### Statistical Analysis

Baseline demographics and clinical characteristics were summarized using descriptive statistics. Continuous variables are shown as mean and standard deviation and categorical variables as frequency and percentage. Results are shown for all study participants and by study group (intervention group A, B, and control group C).

We performed robust linear regression (robreg in Stata), 1-way robust analysis of covariance [18], to analyze how the outcome variables at follow-up differed among treatment groups (intervention with or without counseling and control). We adjusted for baseline measurements of BMI, weight, waist circumference, body fat percentage, and systolic and diastolic blood pressure, and since randomization was done by company and sex, we also adjusted for those variables.

The modern robust approach finds parameter estimates that are less sensitive to outliers and influential data, while at the same time retaining statistical efficiency by minimizing a different target function, which give less weight to individuals with large residuals. The regression coefficients (β) with 95% confidence intervals were estimated based on an iteratively reweighted ordinary least squares algorithm.

We also conducted prespecified subgroup analyses by company where the effect of the intervention between intervention groups and control group (intervention group A vs control group C, intervention group B vs control group C, and intervention group A vs intervention group B) was analyzed.

Missing data was minimal with 99% of participants having complete data on body weight and BMI and 93% having complete data on blood pressure, waist circumference, and body fat percentage. Therefore, all statistical analyses were based on complete cases [19]. The statistical significance was set at *P*<.05. All analyses were completed using Stata.

## Results

In total, 209 participants were recruited to the study. Among these, 4 did not complete baseline measurements and were excluded from all analysis. At the 3-month follow-up, 191 participants had complete data. At baseline, one participant was missing data on weight (1/205, 0.5%), and waist circumference, body fat percentage, and blood pressure were missing from 14 participants (14/205, 6.8%).

Baseline characteristics of all participants divided into the different intervention groups are shown in Table 1. There were 61.5% (126/205) men in the study, 47.8% (98/205) of the participants were office workers, and 52.2% (107/205) were bus drivers. The mean age was 48.3 years. Mean BMI of 27.1 kg/m^2^ indicated overweight, which was in line with a mean waist circumference of 83.1 cm among women and 97.7 cm among men. A waist circumference above 80 cm for women and 94 cm for men is associated with an increased disease risk according to WHO [20]. The mean FINDRISC of 8.6 indicated a somewhat increased risk of developing diabetes type 2 (1/25 individuals compared to 1/100 within 10 years) [21].

**Table 1:** Characteristics of study participants in the Health Integrator study.

		All (n=205)	Intervention group A (n=70)	Intervention group B (n=68)	Intervention group C (n=67)
Age (years), mean (SD)		48.3 (10.0)	48.7 (10.7)	47.9 (10.5)	48.3 (8.8)
**Sex, n (%)**					
	Women	79 (38.5)	27 (38.6)	27 (39.7)	25 (37.3)
	Men	126 (61.5)	43 (61.4)	41 (60.3)	42 (62.7)
BMI (kg/m^2^), mean (SD)		27.1 (4.6)	27.6 (4.6)	26.7 (4.7)	27.2 (4.6)
**Body weight (kg), mean (SD)**					
	Women	72.8 (14.0)	75.9 (16.6)	69.8 (14.0)	72.6 (10.5)
	Men	88.6 (15.8)	87.5 (12.9)	88.4 (16.6)	90.0 (17.9)
**Waist circumference (cm), mean (SD)**					
	Women	83.1 (12.3)	82.3 (10.2)	81.2 (12.2)	85.7 (14.1)
	Men	97.7 (12.9)	97.0 (11.8)	97.1 (13.7)	98.9 (13.4)
**Body fat (%), mean (SD)**					
	Women	35.6 (7.8)	37.0 (8.5)	34.5 (7.0)	35.4 (8.0)
	Men	26.2 (7.1)	25.6 (7.0)	26.4 (7.8)	26.4 (6.6)
**Blood pressure (mm Hg), mean (SD)**					
	Systolic	129 (17)	129 (14)	129 (21)	130 (14)
	Diastolic	82 (10)	81 (8)	82 (12)	82 (8)
**Type of work, n (%)**					
	Office worker	98 (47.8)	31 (44.3)	34 (50.0)	33 (49.3)
	Bus driver	107 (52.2)	39 (55.7)	34 (50.0)	34 (50.7)
**Civil status, n (%)**					
	Partner	163 (79.5)	56 (80.0)	59 (86.8)	48 (71.6)
	No partner	36 (17.6)	10 (14.3)	9 (13.2)	17 (25.4)
**Smoking, n (%)**					
	Yes	18 (8.8)	4 (5.7)	4 (5.9)	10 (14.9)
	No	187 (91.2)	66 (94,3)	64 (94.1)	57 (85.1)
**Snuff use, n (%)**					
	Yes	21 (10.2)	9 (12.9)	3 (4.4)	9 (13.4)
	No	184 (89.8)	61 (87.1)	65 (95.6)	58 (86.6)
**Weekly physical activity (min), n (%)**					
	≥300	64 (31.2)	20 (28.6)	25 (36.8)	19 (28.4)
	150-300	54 (26.3)	19 (27.1)	19 (27.9)	16 (23.9)
	<150	84 (41.0)	29 (41.4)	24 (35.3)	31 (46.37)
**History of treatment, n (%)**					
	Diabetes (yes)	8 (3.9)	3 (4.3)	2 (2.9)	3 (4.5)
	High blood pressure (yes)	26 (12.7)	10 (14.3)	13 (19.1)	3 (4.5)
	High serum lipid levels (yes)	9 (4.4)	5 (7.1)	2 (2.9)	2 (3.0)
FINDRISC^a^, mean (SD)		8.6 (5.3)	9.1 (5.7)	8.2 (5.2)	8.3 (5.0)
HbA_1c_^b^ (mmol/mol), mean (SD)		36.9 (6.1)	37.1 (7.4)	36.8 (5.4)	36.7 (5.3)
HbA_1c_ (%), mean (SD)		5.5 (2.7)	5.5 (2.8)	5.5 (2.6)	5.5 (2.6)
Total cholesterol (mmol/L), mean (SD)		4.85 (1.0)	4.8 (1.0)	4.9 (1.0)	4.9 (0.9)
Apolipoprotein A1 (g/L), mean (SD)		1.43 (0.3)	1.44 (0.3)	1.42 (0.3)	1.44 (0.3)
Apolipoprotein B (g/L), mean (SD)		0.96 (0.3)	0.95 (0.3)	0.97 (0.3)	0.96 (0.2)

^a^FINDRISC: Finnish Diabetes Risk Score.

^b^HbA_1c_: hemoglobin A_1c_.

Results of intervention effects are shown in Table 2. There was a statistically significant intervention effect with intervention group A having a mean waist circumference of approximately 1 cm less compared to the control group at follow-up (β=–0.97, 95% CI –1.84 to –0.10). No other statistically significant differences were seen, although point estimates all favor the intervention group. Comparing intervention group B to the control group, a lower BMI, body weight, waist circumference and body fat percentage were seen at follow-up (BMI: β=–0.35, 95% CI –0.61 to –0.09; body weight: β=–1.08, 95% CI –1.92 to –0.26; waist circumference: β=–1.35, 95% CI –2.24 to –0.45); body fat percentage β=–0.83, 95% CI –1.65 to –0.02). We found no statistically significant difference between group B and the control group with regards to blood pressure. When comparing intervention group A to intervention group B, there was a statistically significant difference in systolic blood pressure (β=–3.74, 95% CI –7.32 to –0.16). No other statistically significant differences were seen when comparing the groups.

**Table 2:** Comparison of intervention effect between intervention groups and control group among all study participants, complete case analysis using robust regression, the Health Integrator study^a^.

	Intervention group A vs control group C, β (95% CI)	Intervention group B vs control group C, β (95% CI)	Intervention group A vs intervention group B, β (95% CI)
BMI (kg/m^2^)	–0.22 (–0.47 to 0.03)	–0.35 (–0.61 to –0.09)	0.13 (–0.13 to 0.38)
Weight (kg)	–0.69 (–1.45 to 0.07)	–1.08 (–1.92 to –0.26)	0.40 (–0.40 to 1.19)
Waist circumference (cm)	–0.97 (–1.84 to –0.10)	–1.35 (–2.24 to –0.45)	0.37 (–0.54 to 1.28)
Body fat (%)	–0.71 (–1.44 to 0.02)	–0.83 (–1.65 to –0.02)	0.12 (–0.65 to 0.88)
SBP^b^ (mm Hg)	–1.24 (–5.01 to 2.53)	2.50 (–1.56 to 6.56)	–3.74 (–7.32 to –0.16)
DBP^c^ (mm Hg)	–0.25 (–2.85 to 2.35)	1.63 (–0.86 to 4.12)	–1.88 (–4.17 to 0.40)

^a^Adjusted for sex, company, and baseline levels of BMI, weight, waist circumference, body fat percentage, and systolic and diastolic blood pressure.

^b^SBP: systolic blood pressure.

^c^DBP: diastolic blood pressure.

Stratified analyses, comparing intervention effects among office workers and bus drivers, are shown in Multimedia Appendix 2. Among office workers, female office workers in intervention group B had a statistically significantly lower body weight and waist circumference compared to women in the control group (body weight: β=–1.66, 95% CI –3.18 to –0.15; waist circumference: β=–1.82, 95% CI –3.11 to –0.52), and male office workers had a lower waist circumference (β=–2.35, 95% CI –3.93 to –0.77). No other differences were seen among office workers when comparing intervention group A or intervention group B with the control group, or when comparing intervention groups A against intervention group B, except that male office workers in intervention group A had a statistically significantly higher waist circumference (β=2.74, 95% CI 0.87 to 4.60) and lower body fat percentage (β=–2.92, 95% CI –0.89 to –0.95) compared to intervention group B after the intervention.

Among bus drivers, BMI at follow-up was lower in both intervention group A (β=–0.38, 95% CI –0.67 to –0.10) and intervention group B (β=–0.45, 95% CI –0.77 to –0.12) compared to the control group. Among male bus drivers in both intervention group A and intervention group B, body weight and waist circumference were statistically significantly lower at follow-up compared to the control group (Group A vs C: body weight: β=–1.13, 95% CI –2.14 to –0.12, waist circumference: β–1.73, 95% CI –3.37 to –0.08, and group B vs C: body weight: β=–1.38, 95% CI –2.54 to –0.22; waist circumference: β=–1.65, 95% CI –3.05 to –0.26). No other statistically significant differences were seen among bus drivers when comparing intervention group A or intervention group B with the control group, or when comparing intervention group A to intervention group B.

## Discussion

### Principal Findings

Our results show that using an mHealth solution such as the Health Integrator smartphone app, focusing on an array of lifestyle habits, may have beneficial effects on health markers of clinical importance, such as weight, waist circumference, and body fat percentage, although the effect sizes were small in this 3-month trial. For overweight persons or persons with obesity, the mean initial 3-month weight loss for the entire sample is too small to be clinically meaningful, but from a public health perspective, small benefits are also appreciable [22].

In our study, those randomized to the group that only met the health coach at baseline and at the end of the study were found to have statistically significant improvements in more clinical variables than those randomized to be in contact with the health coach every 4 weeks. If one group’s intervention is nested within the other (B is within A), then the effect should be expected to be present in group A if it is present in group B, but this was only seen for waist circumference. The reasons for the inconsistent finding raises concerns, and with multiple secondary outcomes, there is a risk of statistical significance by chance.

### Comparison With Previous Findings

The exponential growth of mobile communication has been coupled with an increase in trials using mHealth. Many previous studies have been feasibility studies with relatively few participants [13,23,24]. However, some mHealth studies are comparable in size and scope to ours. The multicomponent mHealth intervention by Spring et al [25] targeting diet and physical activity in 212 North Americans with low fruit and vegetable and high saturated fat intake, low moderate to vigorous activity, and high screen time is similar to our intervention. They reported statistically significant improvements in the intervention groups compared to the controls in terms of both diet and physical activity. They too included regular calls from a trained paraprofessional in addition to using an app. Controls, however, received an app targeting sleep and stress instead of diet and physical activity, while controls in our study received standard care. Intriguingly, having access to a health coach calling twice during our intervention did not lead to better effect of the intervention in terms of clinical outcomes. A similarly designed 3-armed Norwegian mHealth intervention with and without telephone counseling targeting self-management and lifestyle change for persons with type 2 diabetes did not find a better intervention effect among those receiving telephone counseling [12]. Potentially, studies about the mechanisms of change (ie, the processes that operate in behavior change interventions) could be useful to improve delivery of future behavior change interventions.

While weight loss is reasonable to expect in interventions targeting diet, physical activity, or even alcohol habits, previous studies have shown that stress and poor sleep can be important barriers for losing weight [26]. Acute stress tends to increase the preference for sweet food and eating in the absence of hunger, leading to an increased energy intake [27]. This poses the intriguing possibility that the commonly poor outcome of many weight loss programs could benefit from interventions targeting other lifestyle habits that the participants themselves identify as important, other than diet.

### Limitations and Strengths

A limitation to our trial might be the fact that participants were not blinded to their group allocation during the trial. Blinding in randomized trials prevents bias due to, for example, the use of co-interventions or biased ascertainments of outcomes. It is difficult to blind participants in lifestyle interventions. While Spring et al [25] gave the controls an app focusing on sleep and stress instead of the active intervention targeting physical activity and diet, this was not an available option in our study, since we also targeted sleep and stress. Potentially, the controls could have been given a sham lifestyle regimen deemed to be ineffective. For ethical reasons, giving everyone an equal opportunity to improve their lifestyle, we chose to use wait-list controls (ie, after 3 months); when the active intervention was finished, the controls met the health coaches, discussed their health profile, and received access to all offers at the Health Integrator. Although the control group participants were not aware of which offers where included in the app during the first 3 months of the trial, this did not preclude that some controls could have found other ways to improve their lifestyle already during the trial, which would have led to smaller effect sizes.

Blinding of personnel may prevent biased ascertainments of outcome, if the investigator is tempted to look more carefully for certain outcomes in one or the other group. Our laboratory personnel analyzing the blood samples taken were blinded, but other study personnel were not. Nonetheless, clinical variables such as weight, waist circumference, and blood pressure are objective measurers and should be less prone to be biased by unblinded staff.

Social desirability, a natural reaction to defend one’s social image when using self-reported measures, leading to a tendency to overestimate desirable behaviors and underestimate undesirable behaviors [28] may have led to overestimation of, for example, baseline characteristics such as physical activity. Among our participants, 57.5% self-reported meeting the general recommendation of at least 150 minutes of moderate-to-vigorous physical activity per week at baseline. When a population sample of more than a thousand Swedish adults aged 50 to 64 years had their physical activity objectively measured with a hip-worn accelerometer for a week, even more (72.5%) reached the recommendation [29]. It should be kept in mind, however, that wearing an accelerometer may itself affect behavior, the so-called Hawthorne effect. Since all participants in the study also wore an accelerometer at baseline and follow-up, this could have affected behavior among the participants and among the controls. This may be a limitation when evaluating the effect of the intervention, since this potentially led to less difference between groups. It should be noted, however, that the accelerometers did not display any recordings of activity, and none of the participants were informed about their accelerometer data.

A common limitation in intervention studies is dropout and attrition of study participants. In a previous Swedish study evaluating an internet-based weight loss program, only 19.4% (4440/22,860) logged in at least twice during the first 3 months and at least twice during the last 2 months [30]. Nevertheless, compliance in our study was high both in the intervention groups and in the control group. Over 90% of participants had complete data at 3-month follow-up.

While most interventions solely focus on one health behavior such as physical activity [31] or stress [32] or target a specific patient group, such as persons with diabetes [33], pregnant women [34], or patients postsurgery [35], our study had a person-centered approach. The Health Integrator aimed to find the specific lifestyle behavior that the person in question could improve and was interested in improving. This may also have been key to the high compliance in our study. Nevertheless, the fact that participants worked on different types of interventions may have led to power issues in analyses of secondary outcomes. Future researchers may want to include more participants and have longer follow-up.

Another strength of our study is the objective measurement of clinical outcomes, such as waist circumference, body fat percentage, blood pressure, HbA_1c_, and serum lipids. However, the lack of data on HbA_1c_ and serum lipids (ie, total cholesterol, apolipoprotein A1 and apolipoprotein B) at follow-up is a limitation. There were few participants with baseline values outside clinical references and, following clinical practice, only those with pathological values outside the reference were referred for a second blood sampling at the follow-up assessment. Therefore, it was not possible to evaluate effects of the intervention in these markers. Besides, it is unlikely that we would have detected a difference from baseline to follow-up given that the majority of the participants had values within the reference at baseline. However, according to a systematic review including 23 mHealth interventions targeting lifestyle in persons with diabetes, the effect on HbA_1c_ was statistically significant [7].

### Generalizability

Our randomized study included both men and women as well as employees from different types of professions (ie, bus drivers and office workers), potentially representing different socioeconomic groups in society. However, since employment was a prerequisite for participation in our study, this may have created a selection of healthier people than the general population. This selection, which in cohort studies is known as the healthy worker effect, is not an issue in terms of internal validity. However, in terms of external validity, this might translate to an underestimation of the potential efficacy of the intervention. Targeting high-risk unhealthy populations is a well-known way to achieve efficiency and return on investment of an intervention, since there is a greater available risk to be reduced [22].

The recruitment process was slightly different for office workers, who initially got an email from the office of human relations with information about the study, while the study personnel on site in the bus garages orally informed the bus drivers. This could have led to differences in which individuals decided to take part in the study. For example, we know that some bus drivers signed up to get more information about the study together with a colleague. Those participants may have been more compelled to pursue lifestyle changes when they felt supported and connected with colleagues doing the same than if they had signed up alone, which may be more likely when a person receives an email. Peer support during lifestyle intervention has been shown to promote health behavior change [36,37].

The Health Integrator smartphone app was available for both iOS and Android devices. Hence, most smartphone users were able to participate in the study. Nevertheless, the fact that you needed to have access and the ability to handle a smartphone may be a limitation. However, smartphone use in Sweden is widespread, with 92% of the population over age 16 years owning their own smartphone [38]. Thus, the inclusion criteria of having a smartphone is likely not a major limitation in our study setting.

### Conclusion

To conclude, among study participants using only our app that targets the individual’s specific need of lifestyle change, we found small statistically significant differences in body weight, BMI, body fat percentage, and waist circumference after a 3-month intervention when compared to the control group. The effect of additional coaching together with use of the app was unclear.

## Appendix

Multimedia Appendix 1 Study assessments in the Health Integrator randomized controlled trial.

Multimedia Appendix 2 Comparison of intervention effect between intervention groups and control group stratified by type of work; office workers and bus drivers, complete case analysis using robust regression.

Multimedia Appendix 3 CONSORT eHealth Checklist (V1.6.1).

## Abbreviations

EIT: European Institute of Innovation and Technology
FINDRISC: Finnish Diabetes Risk Score
HbA_1c_: glycated hemoglobin A1c
mHealth: mobile health
WHO: World Health Organization

## References

[1] Global Health Observatory data. World Health Organization.

[2] (2013). Global action plan for the prevention and control of noncommunicable diseases. World Health Organization.

[3] (2011). mHealth: New horizons for health through mobile technologies: second global survey on eHealth. World Health Organization.

[4] (2020). How many smartphones are in the world?.

[5] The Swedes and Internet 2018 (Svenskarna och internet 2018).

[6] Lunde P, Nilsson BB, Bergland A, Kværner KJ, Bye A (2018). The effectiveness of smartphone apps for lifestyle improvement in noncommunicable diseases: systematic review and meta-analyses. J Med Internet Res.

[7] Wu X, Guo X, Zhang Z (2019). The efficacy of mobile phone apps for lifestyle modification in diabetes: systematic review and meta-analysis. JMIR Mhealth Uhealth.

[8] Lee M, Lee H, Kim Y, Kim J, Cho M, Jang J, Jang H (2018). Mobile app-based health promotion programs: a systematic review of the literature. Int J Environ Res Public Health.

[9] Villinger K, Wahl DR, Boeing H, Schupp HT, Renner B (2019). The effectiveness of app-based mobile interventions on nutrition behaviours and nutrition-related health outcomes: a systematic review and meta-analysis. Obes Rev.

[10] Schoeppe S, Alley S, Van Lippevelde W, Bray NA, Williams SL, Duncan MJ, Vandelanotte C (2016). Efficacy of interventions that use apps to improve diet, physical activity and sedentary behaviour: a systematic review. Int J Behav Nutr Phys Act.

[11] Wayne N, Perez DF, Kaplan DM, Ritvo P (2015). Health coaching reduces HbA1c in type 2 diabetic patients from a lower-socioeconomic status community: a randomized controlled trial. J Med Internet Res.

[12] Holmen H, Torbjørnsen A, Wahl AK, Jenum AK, Småstuen MC, Arsand E, Ribu L (2014). A mobile health intervention for self-management and lifestyle change for persons with type 2 diabetes, part 2: one-year results from the Norwegian randomized controlled trial RENEWING HEALTH. JMIR Mhealth Uhealth.

[13] Wang J, Cai C, Padhye N, Orlander P, Zare M (2018). A behavioral lifestyle intervention enhanced with multiple-behavior self-monitoring using mobile and connected tools for underserved individuals with type 2 diabetes and comorbid overweight or obesity: pilot comparative effectiveness trial. JMIR Mhealth Uhealth.

[14] Bonn SE, Löf M, Östenson C, Trolle Lagerros Y (2019). App-technology to improve lifestyle behaviors among working adults: the Health Integrator study, a randomized controlled trial. BMC Public Health.

[15] Eysenbach G (2013). CONSORT-EHEALTH: implementation of a checklist for authors and editors to improve reporting of web-based and mobile randomized controlled trials. Stud Health Technol Inform.

[16] Melin J, Bonn SE, Pendrill L, Trolle Lagerros Y (2020). A questionnaire for assessing user satisfaction with mobile health apps: development using Rasch measurement theory. JMIR Mhealth Uhealth.

[17] Martin E, Ruf E, Landgraf R, Hauner H, Weinauer F, Martin S (2011). FINDRISK questionnaire combined with HbA1c testing as a potential screening strategy for undiagnosed diabetes in a healthy population. Horm Metab Res.

[18] Verardi V, Croux C (2009). Robust Regression in Stata. Stata J.

[19] Jakobsen JC, Gluud C, Wetterslev J, Winkel P (2017). When and how should multiple imputation be used for handling missing data in randomised clinical trials: a practical guide with flowcharts. BMC Med Res Methodol.

[20] (2008). Waist circumference and waist-hip ratio: report of a WHO expert consultation. World Health Organization.

[21] Lindström J, Absetz P, Hemiö K, Peltomäki P, Peltonen M (2010). Reducing the risk of type 2 diabetes with nutrition and physical activity: efficacy and implementation of lifestyle interventions in Finland. Public Health Nutr.

[22] Fisher EB, Fitzgibbon ML, Glasgow RE, Haire-Joshu D, Hayman LL, Kaplan RM, Nanney MS, Ockene JK (2011). Behavior matters. Am J Prev Med.

[23] Champion L, Economides M, Chandler C (2018). The efficacy of a brief app-based mindfulness intervention on psychosocial outcomes in healthy adults: a pilot randomised controlled trial. PLoS One.

[24] Plotnikoff RC, Wilczynska M, Cohen KE, Smith JJ, Lubans DR (2017). Integrating smartphone technology, social support and the outdoor physical environment to improve fitness among adults at risk of, or diagnosed with, Type 2 diabetes: findings from the 'eCoFit' randomized controlled trial. Prev Med.

[25] Spring B, Pellegrini C, McFadden HG, Pfammatter AF, Stump TK, Siddique J, King AC, Hedeker D (2018). Multicomponent mHealth intervention for large, sustained change in multiple diet and activity risk behaviors: the make better choices 2 randomized controlled trial. J Med Internet Res.

[26] Geiker NRW, Astrup A, Hjorth MF, Sjödin A, Pijls L, Markus CR (2018). Does stress influence sleep patterns, food intake, weight gain, abdominal obesity and weight loss interventions and vice versa?. Obes Rev.

[27] Rutters F, Nieuwenhuizen AG, Lemmens SGT, Born JM, Westerterp-Plantenga MS (2009). Acute stress-related changes in eating in the absence of hunger. Obesity (Silver Spring).

[28] Warnecke RB, Johnson TP, Chávez N, Sudman S, O'Rourke DP, Lacey L, Horm J (1997). Improving question wording in surveys of culturally diverse populations. Ann Epidemiol.

[29] Ekblom-Bak E, Olsson G, Ekblom ?, Ekblom B, Bergström G, Börjesson M (2015). The daily movement pattern and fulfilment of physical activity recommendations in Swedish middle-aged adults: the SCAPIS pilot study. PLoS One.

[30] van der Mark M, Jonasson J, Svensson M, Linné Y, Rossner S, Lagerros YT (2009). Older members perform better in an internet-based behavioral weight loss program compared to younger members. Obes Facts.

[31] Ek A, Alexandrou C, Söderström E, Bergman P, Delisle Nyström C, Direito A, Eriksson U, Henriksson P, Maddison R, Trolle Lagerros Y, Bendtsen M, Löf M (2020). Effectiveness of a 3-month mobile phone-based behavior change program on active transportation and physical activity in adults: randomized controlled trial. JMIR Mhealth Uhealth.

[32] Bostock S, Crosswell AD, Prather AA, Steptoe A (2018). Mindfulness on-the-go: effects of a mindfulness meditation app on work stress and well-being. J Occup Health Psychol.

[33] Bonn SE, Alexandrou C, Hjörleifsdottir SK, Wiklander K, Östenson C, Löf M, Trolle LY (2018). App-technology to increase physical activity among patients with diabetes type 2: the DiaCert-study, a randomized controlled trial. BMC Public Health.

[34] Henriksson P, Sandborg J, Blomberg M, Alexandrou C, Maddison R, Silfvernagel K, Henriksson H, Leppänen MH, Migueles JH, Widman L, Thomas K, Trolle Lagerros Y, Löf M (2019). A smartphone app to promote healthy weight gain, diet, and physical activity during pregnancy (HealthyMoms): protocol for a randomized controlled trial. JMIR Res Protoc.

[35] Bonn SE, Hult M, Spetz K, Löf M, Andersson E, Wiren M, Trolle Lagerros Y (2020). App technology to support physical activity and intake of vitamins and minerals after bariatric surgery (the PromMera Study): protocol of a randomized controlled clinical trial. JMIR Res Protoc.

[36] Yannitsos D, Murphy RA, Pollock P, Di Sebastiano KM (2020). Facilitators and barriers to participation in lifestyle modification for men with prostate cancer: a scoping review. Eur J Cancer Care (Engl).

[37] Aschbrenner KA, Naslund JA, Bartels SJ (2016). A mixed methods study of peer-to-peer support in a group-based lifestyle intervention for adults with serious mental illness. Psychiatr Rehabil J.

[38] The Swedes and Internet 2019 (Svenskarna och internet 2019).

