# Metadata Correction: Clinical Outcomes Among Working Adults Using the Health Integrator Smartphone App: Analyses of Prespecified Secondary Outcomes in a Randomized Controlled Trial

**DOI:** 10.2196/38199

**Published:** 2022-03-24

**Authors:** Stephanie Bonn, Gabriella Licitra, Rino Bellocco, Ylva Trolle Lagerros

**Affiliations:** 1 Clinical Epidemiology Division Department of Medicine Solna Karolinska Institutet Stockholm Sweden; 2 Department of Medical Epidemiology and Biostatistics Karolinska Institutet Stockholm Sweden; 3 Department of Statistics and Quantitative Methods University of Milano-Bicocca Milan Italy; 4 Center for Obesity, Academic Specialist Center Stockholm Health Services Stockholm Sweden

In “Clinical Outcomes Among Working Adults Using the Health Integrator Smartphone App: Analyses of Prespecified Secondary Outcomes in a Randomized Controlled Trial” (J Med Internet Res 2022;24(3):e24725), the following correction was made:

In the originally published article, the names of peer reviewers’ names inadvertently included an additional reviewer.

The correct statement of peer review should be “*peer-reviewed by X Guo*.”

The correction will appear in the online version of the paper on the JMIR Publications website on March 24, 2022, together with the publication of this correction notice. Because this was made after submission to PubMed, PubMed Central, and other full-text repositories, the corrected article has also been resubmitted to those repositories.

