# Supplementary material for: Clinical Outcomes Among Working Adults Using the Health Integrator Smartphone App: Analyses of Prespecified Secondary Outcomes in a Randomized Controlled Trial
Source: J Med Internet Res. 2022 Mar 21;24(3):e24725. doi: 10.2196/24725 (PMC8946520; doi:10.2196/24725)
Supplement: Multimedia Appendix 1 [file jmir_v24i3e24725_app1.docx]

**Multimedia Appendix 1. Study assessments in the Health Integrator randomized controlled trial.**

| Method | This was assessed | Tool |
| --- | --- | --- |
| **Clinical measurements** | Body composition | Body weight (kg), fat percentage, and skeletal muscle percentage measured with OMRON BF 511 |
|  | Waist circumference |  |
|  | Height |  |
|  | Blood pressure |  |
|  | Pulse |  |
|  | Hemoglobin A1c (HbA1c) |  |
|  | Total cholesterol |  |
|  | Apolipoprotein A1 |  |
|  | Apolipoprotein B |  |
| **Accelerometer measured physical activity** | Physical activity/inactivity | ActiGraph wGT3x-BT |
| **Questionnaires** | Participant characteristics |  |
|  | Diabetes risk | The Finnish Diabetes Risk Score (FINDRISC) |
|  | Sleep quality and restoration from sleep | Karolinska Sleep Questionnaire (KSQ) |
|  | Dietary intake | Semi-quantitative food frequency questionnaire (FFQ) |
|  | Eating patterns | Three Factor Eating Questionnaire (TFEQ-R21) |
|  | Physical activity and inactivity | Two questions developed for clinical practice by the Swedish National Board of Health and Welfare |
|  | Health Related Quality of Life | RAND-36 ^a^ |
|  | Purpose in Life | Life Engagement Test |
|  | Perceived stress levels | Perceived Stress Scale (PSS-14) |
|  | Social support for a healthy lifestyle | 6-item questionnaire about support from family, friends and coworkers |
|  | Stage-of-change, motivation to make lifestyle changes | Regarding diet, physical activity, sleeping habits, stress, alcohol and tobacco |

^a^ RAND-36 includes 36 questions (items) about physical and mental health, whereof 35 cover eight health concepts: physical functioning, role limitations caused by physical health problems, role limitations caused by emotional problems, social functioning, emotional well-being, energy/fatigue, bodily pain, and general health perception.
