# Supplementary material for: Clinical Outcomes Among Working Adults Using the Health Integrator Smartphone App: Analyses of Prespecified Secondary Outcomes in a Randomized Controlled Trial
Source: J Med Internet Res. 2022 Mar 21;24(3):e24725. doi: 10.2196/24725 (PMC8946520; doi:10.2196/24725)
Supplement: Multimedia Appendix 2 [file jmir_v24i3e24725_app2.docx]

| **Appendix 2**. Comparison of intervention effect between intervention groups and control group stratified by type of work; office workers and bus drivers, complete case analysis using robust regression. | | | | | | | |
| --- | --- | --- | --- | --- | --- | --- | --- |
|  | | **Intervention group A**  **vs. Control group C** | | **Intervention group B**  **vs. Control group C** | | **Intervention group A**  **vs. Intervention group B** | |
| **OFFICE WORKERS** | |  |  |  |  |  |  |
|  | | **β** | **(95% CI)** | **β** | **(95% CI)** | **β** | **(95% CI)** |
| **BMI**, kg/m^2^ | | -0.04 | (-0.46 to 0.38) | -0.22 | (-0.71 to 0.27) | 0.18 | (-0.25 to 0.61) |
| **SBP**, mmHg | | -1.07 | (-7.55 to 5.41) | 1.84 | (-4.03 to 7.71) | -2.91 | (-7.54 to 1.73) |
| **DBP**, mmHg | | 0.11 | (-3.96 to 4.19) | 2.85 | (-0.94 to 6.64) | -2.74 | (-5.70 to 0.21) |
| **Body weight**, kg | |  |  |  |  |  |  |
|  | All | -0.07 | (-1.29 to 1.16) | -0.67 | (-2.07 to 0.73) | 0.60 | (-0.65 to 1.86) |
|  | Women | -0.89 | (-2.16 to 0.39) | **-1.66** | **(-3.18 to -0.15)** | 0.78 | (-0.51 to 2.06) |
|  | Men | 1.86 | (-0.46 to 4.19) | 1.87 | (-0.08 to 3.82) | -0.00 | (-2.38 to 2.37) |
| **Waist circumference**, cm | |  |  |  |  |  |  |
|  | All | -0.49 | (-1.56 to 0.57) | **-1.56** | **(-2.71 to -0.41)** | 1.07 | (-0.12 to 2.26) |
|  | Women | -0.85 | (-2.16 to 0.46) | **-1.82** | **(-3.11 to -0.52)** | 0.97 | (-0.32 to 2.25) |
|  | Men | 0.39 | (-1.62 to 2.40) | **-2.35** | **(-3.93 to -0.77)** | **2.74** | **(0.87 to 4.60)** |
| **Body fat percent**, % | |  |  |  |  |  |  |
|  | All | -0.54 | (-1.73 to 0.65) | -0.55 | (-1.88 to 0.78) | 0.01 | (-1.19 to 1.21) |
|  | Women | -0.63 | (-1.92 to 0.67) | -1.40 | (-3.03 to 2.45) | 0.77 | (-0.55 to 2.08) |
|  | Men | -0.89 | (-3.03 to 1.26) | 1.38 | (-0.90 to 3.66) | **-2.27** | **(-3.89 to -0.65)** |
|  |  |  |  |  |  |  |  |
| **BUS DRIVERS** | |  |  |  |  |  |  |
|  | | **β** | **(95% CI)** | **β** | **(95% CI)** | **β** | **(95% CI)** |
| **BMI**, kg/m^2^ | | **-0.38** | **(-0.67 to -0.10)** | **-0.45** | **(-0.77 to -0.12)** | 0.07 | (-0.28 to 0.41) |
| **SBP**, mmHg | | -2.76 | (-9.05 to 3.54) | 2.45 | (-3.37 to 8.26) | -5.20 | (-11.57 to 1.16) |
| **DBP**, mmHg | | -0.84 | (-4.35 to 2.67) | -0.34 | (-3.02 to 3.69) | -1.18 | (-5.21 to 2.86) |
| **Body weight**, kg | |  |  |  |  |  |  |
|  | All | **-1.19** | **(-2.09 to -0.28)** | **-1.29** | **(-2.35 to -0.24)** | 0.11 | (-0.98 to 1.19) |
|  | Women^*^ | - | - | - | - | - | - |
|  | Men | **-1.13** | **(-2.14 to -0.12)** | **-1.38** | **(-2.54 to -0.22)** | 0.26 | (-1.00 to 1.51) |
| **Waist circumference**, cm | |  |  |  |  |  |  |
|  | All | **-1.67** | **(-3.30 to -0.05)** | -1.26 | (-2.63 to 0.11) | -0.41 | (-1.80 to 0.97) |
|  | Women* | - | - | - | - | - | - |
|  | Men | **-1.73** | **(-3.37 to -0.08)** | **-1.65** | **(-3.05 to -0.26)** | -0.07 | (-1.53 to 1.39) |
| **Body fat percent**, % | |  |  |  |  |  |  |
|  | All | -0.85 | (-1.80 to 0.10) | -0.93 | (-1.88 to 0.03) | 0.07 | (-0.98 to 1.13) |
|  | Women* | - | - | - | - | - | - |
|  | Men | -0.98 | (-2.02 to 0.07) | -1.18 | (-2.50 to 0.13) | 0.20 | (-1.34 to 1.75) |
| ^*^Too few observations (n=8) to perform analysis. BMI, Body Mass Index; SBP, Systolic Blood Pressure; DBP, Diastolic Blood Pressure | | | | | | | |
